# A Scoring Model Using Multi-Metabolites Based on Untargeted Metabolomics for Assessing Dyslipidemia in Korean Individuals with Obesity

**DOI:** 10.3390/metabo15040279

**Published:** 2025-04-17

**Authors:** Su-Geun Yang, Hye Jin Yoo

**Affiliations:** 1 Department of Biomedical Science, BK21 FOUR Program in Biomedical Science and Engineering, Inha University, Incheon 22332, Republic of Korea; su-geun.yang@inha.ac.kr; 2 Inha Institute of Aerospace Medicine, Inha University College of Medicine, Incheon 22332, Republic of Korea; 3 Institute for Specialized Teaching and Research (INSTAR), Inha University, Incheon 22332, Republic of Korea

**Keywords:** metabolite risk score, dyslipidemia, metabolomics, Korean population

## Abstract

Background/Objectives: Metabolite risk score (MRS), which considers the collective effects of metabolites closely reflecting a phenotype, is a new approach for disease assessment, moving away from focusing solely on individual biomarkers. This study aimed to investigate a metabolite panel for dyslipidemia and verify the diagnostic efficacy of MRS on dyslipidemia. Methods: Key metabolite identification and MRS establishment were conducted in the discovery set, and MRS validation was performed in the replication set, with 50 healthy individuals and 50 dyslipidemia patients in each set. The MRS was constructed using key metabolites, identified via UPLC-MS/MS analysis, employing a weighted approach based on linear regression analysis. Results: N-acetylisoputreanine-γ-lactam and eicosapentaenoic acid were identified as key metabolites for dyslipidemia and were utilized for establishing the MRS. In addition to the MRS model, a conventional dyslipidemia diagnostic model based on lipid profiles, as well as a combined model (MRS + lipid profiles), were also established. In the discovery set, the MRS model diagnosed dyslipidemia with 85.4% accuracy. When combined with lipid profiles, accuracy improved to 91.8%. In the replication set, the MRS demonstrated diagnostic power with 76.1% accuracy, while the combined model achieved 86.0% accuracy for dyslipidemia assessment. Conclusions: The MRS alone indicated sufficient assessment power in a real-world setting, despite a slight reduction in assessment ability when validated in the replication set. At this stage, therefore, the MRS serves as an auxiliary tool for disease diagnosis. This first attempt to apply MRS for dyslipidemia may offer a foundational concept for MRS in this disease.

## 1. Introduction

Dyslipidemia is a chronic disease that shows elevated levels of triglyceride (TG), low-density lipoprotein-cholesterol (LDL-C), and total cholesterol (TC) and reduced levels of high-density lipoprotein-cholesterol (HDL-C) in the blood. The incidence of dyslipidemia is closely linked to the risk of vascular diseases such as atherosclerotic events and stroke and is often observed with obesity and type 2 diabetes simultaneously as well [1,2,3]. According to the dyslipidemia fact sheet in Korea published in 2022, the mean prevalence of dyslipidemia from 2016 to 2020 was 48.2%, indicating a quite high proportion within the population [1]. Moreover, individuals with type 2 diabetes, hypertension, and obesity are accompanied by dyslipidemia at a ratio of 87.1%, 72.1%, and 55.4%, respectively [1]. Given that lipid disorders and subsequent events, including coronary artery disease and ischemic stroke, are regarded as leading causes of premature death even globally [3], it is crucial to monitor and manage dyslipidemia effectively.

Metabolites serve as biosignatures that closely mirror an individual’s phenotypic information since they are downstream products of metabolic processes [4]. Abnormalities in metabolism can lead to alterations in metabolites, making their accumulation or deficiency indicative hallmarks of diseases [4]. Hence, metabolomics has been presented as a promising tool for investigating diseases’ biomarkers and status. Researchers have attempted to elucidate what novel biomarkers are for dyslipidemia and the biological meaning of altered metabolites observed in patients with dyslipidemia via metabolomics study. Du et al. [5] revealed 69 plasma metabolites indicating disturbed regulation of inflammation and lipid metabolism in dyslipidemia patients; notably, indole-3-propionic acid particularly emerged as a promising biomarker. Ke et al. [6] demonstrated that 46 metabolites implicated in macrophage activation and oxidized (ox-) LDL formation were identified as potential biomarkers for dyslipidemia in the UK population. Wang et al. [7] showed a positive association between plasma retinol levels and dyslipidemia in the Chinese population.

Like previous studies, most research has been focused on investigating metabolic pattern changes and identifying the best novel biomarkers for a specific disease. However, diseases arise from the interactions between metabolites significantly related to the disease. In this context, metabolite risk score (MRS), which considers the comprehensive effects among metabolites, can be a novel concept for approaching disease progression. So far, a limited number of studies have investigated MRS. A study reported that future weight gain could be predicted by constructing an MRS using 8 metabolites [8]. Another recent study validated that an adipose tissue-derived MRS, incorporating hundreds of metabolites, aids in assessing the risk of type 2 diabetes before the elevation of fasting glucose levels [9]. Furthermore, mild cognitive impairment was found to be predictable based on MRS established by 61 metabolites [10]. The MRS studies indicated that this scoring system demonstrates improved assessment capability for diseases. Hence, the development of MRS is crucial to prevent future disease onset.

To date, there has been no research on MRS for dyslipidemia. Therefore, the present study aimed to establish and validate an MRS for the diagnosis of dyslipidemia in a Korean population by screening significant metabolites via ultra-performance liquid chromatography (UPLC)-tandem mass spectrometry (MS/MS). The decision to focus on MRS as a diagnostic tool was based on two key considerations. First, current standard diagnostic criteria for dyslipidemia have certain limitations. For instance, the Friedewald equation, which is commonly used in clinical settings due to its cost-effectiveness and convenience, becomes inaccurate when TG levels exceed 400 mg/dL. In addition, standard blood tests are unable to measure highly atherogenic subtypes such as small dense LDL. Second, since our cohort is cross-sectional in design, a diagnostic approach is more methodologically appropriate than a predictive one. Finally, this study contributes to the advancement of novel approaches to the use of MRS in dyslipidemia diagnosis.

## 2. Materials and Methods

### 2.1. Collection of Plasma Samples and Study Design

A total of 100 fasting plasma samples from healthy individuals and another 100 fasting plasma samples from dyslipidemia patients were acquired through the Korea Biobank Network, including the Biobank of Korea-Chungbuk National University Hospital (CBNUH) and the Biobank Ajou University Hospital under the institutional review board (IRB)-approved protocols (Inha University IRB, approved No.: 21004-1AR). The IRB waived the requirement to obtain written informed consent from study participants, as all samples and data were procured from the Biobanks with explicit authorization. The Biobanks randomly selected samples from their pools based on the following inclusion criteria: (1) Korean males and females, (2) aged between 40 and 60, and (3) Korean Standard Classification of Diseases (KCD) code Z00 for healthy individuals, and (4) KCD code E78.2 and E78.5 for dyslipidemia patients. The general information was also collected as well: age, sex, body weight, body mass index (BMI), systolic/diastolic blood pressures (BPs), and fasting levels of glucose, lipid profiles [TG, HDL-C, LDL-C, TC], aspartate aminotransferase (AST), alanine aminotransferase (ALT), and γ-glutamyltransferase (γ-GTP).

**Figure 1:**
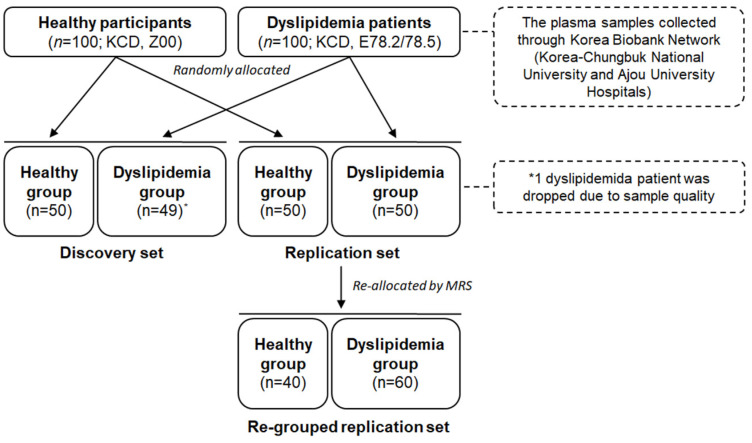
Flow chart of the study. * One dyslipidemia patient in the discovery set was excluded due to poor sample quality after random allocation.

This study was designed as a cross-sectional, two-phase approach comprising a biomarker discovery stage (discovery) and an independent validation stage (replication). As illustrated in Figure 1, each of the two groups (healthy and dyslipidemia; *n* = 100 each) was further randomly divided into a discovery set and a replication set in a 1:1 ratio. This sample size was determined using G*Power ver. 3.1.9.7 (Franz Faul, University of Kiel, Kiel, Germany), which achieved a statistical power of 0.8 with an α error probability of 0.05 and an effect size exceeding 0.5.

Key metabolites were identified and used to establish the MRS in the discovery set. Subsequently, the diagnostic performance of the MRS for dyslipidemia was validated in the replication set. The replication set was re-allocated based on the MRS (re-grouped replication set) to further assess its accuracy in distinguishing actual disease conditions, and the results were compared with those of the original replication set (Figure 1).

### 2.2. Biochemical Analysis

Inflammatory and oxidative stress conditions were additionally evaluated using cytokines [interleukin (IL)-1β, IL-6, tumor necrosis factor (TNF)-α, interferon (IFN-γ)] and ox-LDL. Cytokines were analyzed by commercial kits, Human High Sensitivity T cell Magnetic Bead Panel (Merck Millipore, Billerica, MA, USA), and the resulting reactions were analyzed by a MAGPIX system (Luminex Corporation, Austin, TX, USA). Ox-LDL was assessed using Oxidized LDL kits (Mercodia, Uppsala, Sweden), and the resulting reactions were detected by SpectraMax 190 (Molecular Devices Corp., Sunnyvale, CA, USA).

### 2.3. Untargeted Metabolomics

The 100 μL plasma samples were combined with 300 μL of 70% cold acetonitrile (Thermo Fisher Scientific, Fair Lawn, NJ, USA). After vortexing, the mixtures were incubated (4 °C, 10 min). Subsequently, the mixtures were centrifuged at 13,000 rpm for 15 min at 4 °C. The supernatant was collected and transferred to a new vial, followed by drying using SpeedVac (Gyrozen, Gyeonggi-do, Republic of Korea). 10% methanol (J.T.Baker^®^; Avantor, Radnor, PA, USA) containing internal standards [ISTDs; L-leucine-^13^C_6_ (Sigma-Aldrich, St. Louis, MO, USA) and stearic-d_35_ acid (Sigma-Aldrich, St. Louis, MO, USA)] was utilized to re-dissolve the dried residues. Pooled plasma samples for quality control purposes were prepared using the same procedure.

5 μL of the prepared samples were injected into the LC system [Acquity UPLC-BEC-C18 column (2.1 × 10 mm, 1.7 μm); Waters, Milford, MA, USA] equipped with Ultimate 3000 RSLC System (Thermo Fisher Scientific, Bremen, Germany). Quality control samples were injected every 10th run. Mobile phases A and B were 0.1% formic acids (Merck Millipore, Billerica, MA, USA) dissolved in LC-MS grade water (Thermo Fisher Scientific, Fair Lawn, NJ, USA) and acetonitrile (Thermo Fisher Scientific, Fair Lawn, NJ, USA), respectively. Changes in the gradient of the mobile phases ranging from 0% to 100% were applied over 17 min at a flow rate of 0.4 mL/min. MS analysis was conducted using electrospray ionization (ESI) in both positive and negative modes with a Q-Exactive Orbitrap Plus MS (Thermo Fisher Scientific, Waltham, MA, USA). A full MS-ddms2 scan type [80~1000 mass-to-charge (m/z)] was employed. The following MS conditions were used: spray voltage (kV), 3.5; sheath gas flow rate, 50 arbitrary units; auxiliary gas, 13 arbitrary units; S-lens radio frequency level, 55; and capillary temperature, 370 °C.

### 2.4. Establishment of MRS and Its Validation

#### 2.4.1. Major and Key Metabolites Related to Dyslipidemia in the Discovery Set

To select major metabolites, identified metabolites were screened based on variable importance in projection (VIP) value of ≥1.5 and false rate discover (FDR)-adjusted *p*-value (*q*-value) of <0.05 in the discovery set. These major metabolites were further screened to find out key metabolites associated with dyslipidemia through the following methods: first, a linear regression analysis using the stepwise approach was employed to verify dyslipidemia-related metabolites; and then, the verified metabolites were assessed using a receiver operating characteristic (ROC) curve analysis to evaluate their assessment ability for dyslipidemia. Finally, metabolites demonstrating significance in the ROC curve analysis were determined as key metabolites and utilized for establishing MRS.

#### 2.4.2. MRS Establishment in the Discovery Set

The key metabolites were converted into MRS using a weighted approach, establishing the equation of the MRS as ∑*β_i_M_i_*, where *β_i_* represents the value of standardized β-coefficient and *M_i_* represents the simple score of each key metabolite. A linear regression analysis with the enter method was employed to obtain the standardized β-coefficient values. Subsequently, ROC curve analysis was performed to determine the cut-off values of the key metabolites, converting their relative intensities into a simple score; if the relative intensity was above the cut-off value, the simple score was 1; otherwise, it was 0.

#### 2.4.3. MRS Validation in Both Discovery and Replication Set

The MRS model, individual key metabolite models, and a lipid profile model were constructed and compared with each other within the discovery set to evaluate the assessment ability of the MRS for dyslipidemia. Note that the lipid profile model consisted of TG, HDL-C, LDL-C, and TC, which are conventional markers for dyslipidemia. Then, the models were validated to determine whether the assessment power of these models remained consistent in the replication set.

### 2.5. Statistical Analysis

The details are provided in the supplementary information. Briefly, for putative metabolite identification and relative peak intensity data acquisition, Compound Discoverer ver. 3.3 SP 2 software (Thermo Fisher, Waltham, MA, USA) was used. SIMCA 17 software (Sartorius-Umetrics, Göttingen, Germany) was utilized for orthogonal partial least squares-discriminant analysis (OPLS-DA), loading plot generation, permutation tests, and VIP score analysis.

Statistical analysis was conducted using IBM SPSS Statistics 28.0 (IBM Corp., Armonk, NY, USA). This included the comparison of variables between groups, linear regression analysis, and ROC curve analysis. A significance threshold of two-tailed *p* < 0.05 was used for all analyses. For the comparison of metabolite relative intensities, meanwhile, *q*-values were additionally computed to adjust for FDR, using the R software ver. 4.4.2 (R Foundation for Statistical Computing, Vienna, Austria) with package fdrtool ver. 1.2.18 [https://CRAN.R-project.org/package=fdrtool (accessed on 1 November 2024)], with significance defined as *q* < 0.05 to mitigate type 1 error.

## 3. Results

### 3.1. General Clinical/Biochemical Markers Between the Groups in the Discovery Set

As shown in Table 1, individuals in the dyslipidemia group were significantly older than those in the healthy group. Additionally, both body weight and BMI were significantly higher in the dyslipidemia group than in the healthy group. Although the sex distribution was not equal between the groups, it did not show a statistically significant difference (Table 1). While sex differences may influence body weight due to physical and physiological differences between males and females, the presence of only three more males and four fewer females in the dyslipidemia group is unlikely to account for the observed 7.1 kg difference in average body weight. Therefore, the increase in body weight primarily reflects the metabolic characteristics of the dyslipidemia group rather than being driven solely by sex distribution. Consequently, the three variables that showed significant differences between the groups (i.e., age, body weight, and BMI) were used as confounding factors for adjustment. After adjusting for these confounding factors, systolic BP, glucose levels, and TNF-α retained their statistical significance, and diastolic BP became significantly elevated. Of note, individuals in the dyslipidemia group appear to exhibit early-stage hypertension, a well-known complication associated with dyslipidemia. In contrast, the statistical significance of TG, TC, AST, ALT, IL-1β, IL-6, and ox-LDL disappeared after adjustment.

### 3.2. UPLC-MS/MS Analysis

In the ESI positive and negative modes, a total of 452 and 258 metabolites, respectively, were detected and putatively identified. Among these, unidentified metabolites and drugs were excluded. Finally, 128 and 69 metabolites in ESI positive and negative modes remained, respectively, and utilized for the subsequent analysis (Table S1).

#### 3.2.1. Metabolic Characteristics Between the Healthy and Dyslipidemia Groups in the Discovery Set

**Figure 2:**
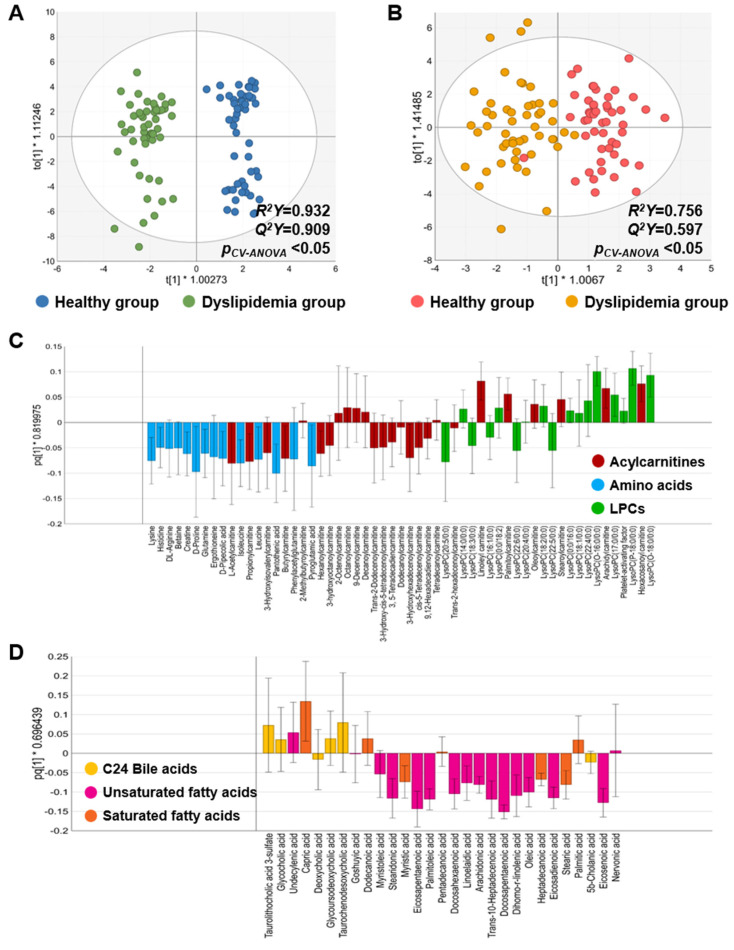
OPLS-DA and loading plots of the positive and negative modes in the discovery set. (**A**,**C**) OPLS-DA and loading plots in the positive modes, respectively. The top 3 abundant metabolite subclasses observed in the positive mode (acylcarnitine, amino acids, and LPCs) were used for creating the loading plot. (**B**,**D**) OPLS-DA and loading plots in the negative modes, respectively. The top 3 abundant metabolite subclasses observed in the negative mode (C24 bile acids, unsaturated fatty acids, and saturated fatty acids) were used for creating the loading plot.

In both ESI positive and negative modes, the OPLS-DA results indicated that the groups significantly discriminated with enough *R*^2^*Y* and *Q*^2^*Y* values (both *R*^2^*Y* and *Q*^2^*Y* >0.5) and p*_CV-ANOVA_* values less than 0.05 (Figure 2A,B). The permutation test results also demonstrated group discrimination without overfitting in both modes (Figure S1). In ESI positive mode, the three most abundant subclasses of the detected metabolites were acylcarnitines (ACs), amino acids, and lysophosphatidylcholines (LPCs); thus, a loading plot was consequently generated using these three metabolites’ subclasses (Figure 2C). As shown in the plot, a distinct pattern of the metabolites was observed; particularly, most of short-chain ACs (L-acetylcarnitine, propionylcarnitine, 3-hydroxyisovalerylcarnitine, butyrylcarnitine, and hexanoylcarnitine) and amino acids were prevalent in the dyslipidemia group, whereas long-chain ACs with acyl chains over C18 (palmitoylcarnitine, linoleylcarnitine, oleoylcarnitine, stearoylcarnitine, arachidylcarnitine, hexacosanoylcarnitine) were plenty in the healthy group (Figure 2C). In the ESI negative mode, a loading plot was created based on the top 3 abundant metabolites’ subclasses, including C24 bile acids, unsaturated fatty acids (UFAs), and saturated fatty acids (SFAs) (Figure 2D). Similar to the ESI positive mode, a clearly different pattern of the metabolites was also observed in ESI negative mode; most of the UFAs and SFAs showed elevated levels in the dyslipidemia group (Figure 2D). Based on the results, we confirmed that altered metabolic patterns distinguish between the healthy and dyslipidemia groups.

#### 3.2.2. Major Metabolite Screening and Key Metabolite Selection

**Figure 3:**
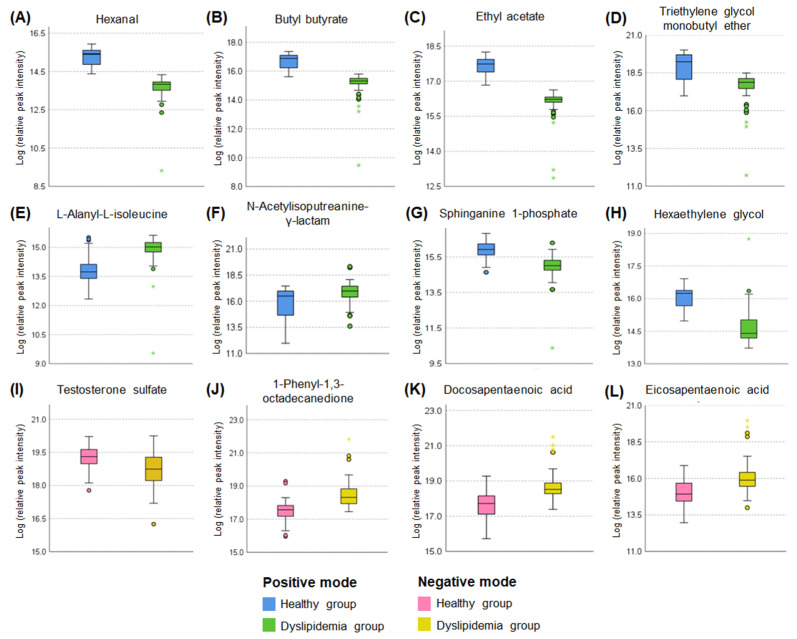
Box plots of the major metabolites (VIP ≥ 1.5 and *q* < 0.05) of positive and negative modes in the discovery set. Mean±standard error (SE). Following logarithmic transformation, independent *t*-tests were performed to compare the healthy and dyslipidemia groups. The false discovery rate (FDR) was adjusted to present *q*-values for correcting multiple comparison errors of metabolites; *q*-value < 0.05 is considered to be significant. (**A**–**H**) The metabolites have both VIP score ≥ 1.5 and *q* < 0.05 in the positive mode. (**I**–**L**) The metabolites have both VIP score ≥ 1.5 and *q* < 0.05 in the negative mode. Circles and asterisks indicate mild and extreme outliers, respectively.

According to the VIP score analysis, a total of 11 and 4 metabolites had a VIP score of over 1.5 in ESI positive and negative modes, respectively (Figure S2). Most of them had significant *q*-values (*q* < 0.05) except for 3 metabolites in ESI positive mode, resulting in 12 metabolites being selected as major metabolites (Figure 3).

In the ESI positive mode, levels of six metabolites (hexanal, butyl butyrate, ethyl acetate, triethylene glycol monobutyl ether, sphinganine 1-phosphate, and hexaethylene glycol) were decreased, while levels of two metabolites (L-alanyl-L-isoleucine and N-acetylisoputreanine-γ-lactam) were increased in the dyslipidemia group compared to the healthy group (Figure 3A–H). In the ESI negative mode, one metabolite (testosterone sulfate) showed a lower level, whereas three metabolites [1-phenyl-1,3-octadecanedione, docosapentaenoic acid (DPA), and eicosapentaenoic acid (EPA)] exhibited higher levels in the dyslipidemia group than in the healthy group (Figure 3I–L).

Using the 12 major metabolites, linear regression analysis with a stepwise method was performed to select notable dyslipidemia-related key metabolites. Among them, hexanal and butyl butyrate were ruled out because of multicollinearity problems during the analysis. As a result, ethyl acetate, hexaethylene glycol, N-acetylisoputreanine-γ-lactam, and EPA emerged as candidate metabolites. Consequently, ROC analysis revealed that two metabolites, N-acetylisoputreanine-γ-lactam and EPA, displayed significant diagnostics ability for dyslipidemia, thereby being selected as key metabolites for constructing MRS.

### 3.3. Establishment of MRS and Its Assessment Ability for Dyslipidemia

As mentioned above, N-acetylisoputreanine-γ-lactam and EPA were utilized for MRS establishment. To score the two metabolites, first, cut-off values (relative peak intensities) of them were obtained through Youden’s index method (N-acetylisoputreanine-γ-lactam: 17622907.3327901; and EPA: 45400161.1054486). If the relative peak intensities of the metabolites were above the cut-off values, they were simply scored as 1; otherwise, they were simply scored as 0. Then, the weight values (standardized β from linear regression analysis) of each metabolite were determined, as shown in Table S2, and were used to multiply the simple score. Consequently, the equation of the MRS, the final scoring, is as follows: ∑*β_i_M_i_*, where *β_i_* and *M_i_* represent the value of standardized β and simple score of each key metabolite, respectively.

**Figure 4:**
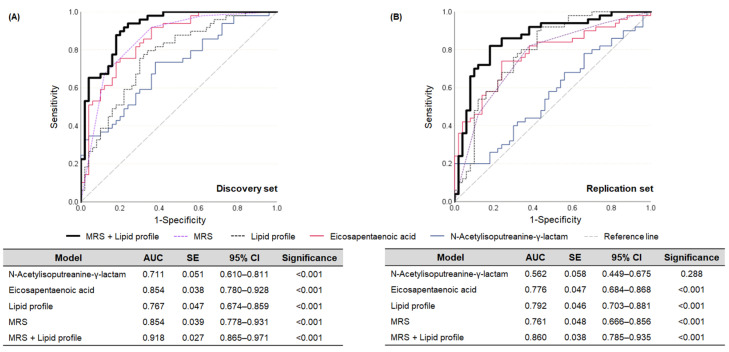
Prediction abilities of the key metabolites, lipid profiles, MRS, and combination of MRS and lipid profiles for dyslipidemia. MRS was calculated in the discovery set following the equation: ∑*β_i_M_i_*, where *β_i_* is a beta weight of a linear regression model comprising the two key metabolites (N-acetylisoputreanine-γ-lactam and eicosapentaenoic acid), and *M_i_* is a simple score (0 or 1) of the two key metabolites according to their cut-off value. The weights and cut-off values for simple scoring of the key metabolites were applied without modification in the replication set to calculate the MRS. A lipid profile model was established with TG, HDL-C, LDL-C, and TC. (**A**) Comparison of the models in the discovery set. (**B**) Comparison of the models in the replication set. AUC: area under the curve. CI: confidence interval. HDL: high-density lipoprotein. LDL: low-density lipoprotein. MRS: metabolite risk score. SE: standard error. TC: total cholesterol. TG: triglyceride.

Additional diagnostics models were established to compare the assessment ability of the MRS on dyslipidemia. These models consisted of each metabolite, the lipid profile (TG, HDL-C, LDL-C, and TC), and a combination of the MRS and lipid profile. In Figure 4A, in the discovery set, the MRS model significantly diagnosed dyslipidemia with an accuracy of 85.4%, demonstrating enhanced assessment ability compared to the lipid profile, the conventional diagnostic factor, which had an accuracy of 76.7%. In addition, the combination model of the MRS and lipid profile showed the highest assessment ability with 91.8% accuracy, suggesting the potential of the MRS as an adjuvant tool.

These models were locked down and applied to the replication set to verify whether their assessment ability remained intact. Unfortunately, the assessment ability of the MRS decreased by about 3% compared to that of the lipid profile, although statistical significance remained (Figure 4B). This might be attributed to the reduced assessment ability of N-acetylisoputreanine-γ-lactam (56.2%) (Figure 4B). In other words, this metabolite was not effectively applicable for use in the other independent population. Nevertheless, EPA exhibited sufficient assessment ability for dyslipidemia, and the combination model (MRS + lipid profile) was validated as having enhanced assessment ability compared to the conventional model (lipid profile) (Figure 4B). These results may emphasize the role of MRS as an assistant tool for dyslipidemia diagnostics.

### 3.4. Discrimination Performance of the MRS in the Re-Grouped Replication Set

**Figure 5:**
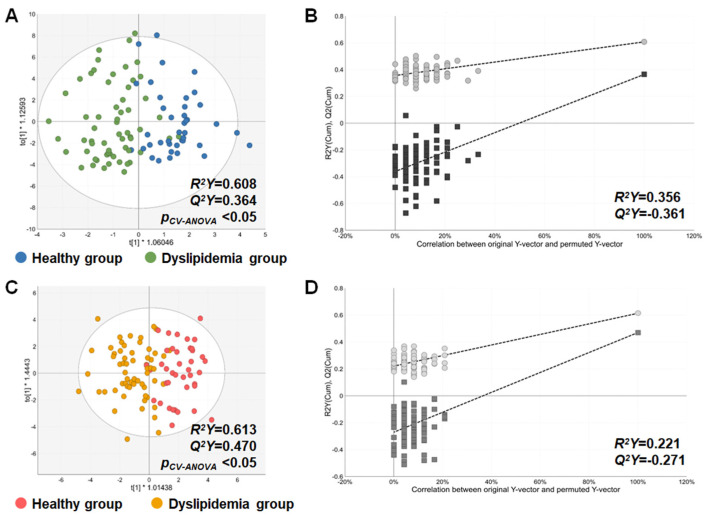
OPLS-DA and permutation test plots of the positive and negative modes in the re-grouped replication set based on the MRS. The individuals in the replication set were re-assigned into the healthy and dyslipidemia groups divided according to the MRS computed by the equation established from the discovery set; the healthy group was MRS < [MRS]*_median_*, and the dyslipidemia group was MRS ≥ [MRS]*_median_*. (**A**,**B**) OPLS-DA and permutation test plots in the positive modes, respectively. (**C**,**D**) OPLS-DA and permutation test plots in the negative modes, respectively.

We investigated how accurately patients could be classified when applying the MRS to actual practice. The replication set was re-grouped into healthy and dyslipidemia groups according to the MRS. Individuals with an MRS less than its median value ([MRS]*_median_*: 0.520) were re-assigned to the healthy group (*n* = 40), while those with an MRS equal or greater than the [MRS]*_median_* were included in the dyslipidemia group (*n* = 60). Of the individuals, 31 from the healthy group (77.5%) and 41 from the dyslipidemia group (68.3%) were correctly identified as healthy individuals and dyslipidemia patients, respectively (Table S3), suggesting that MRS can be utilized as an auxiliary tool for assessing disease status. In addition, the MRS was deemed effective in discriminating between the groups, as evidenced by significant p*_CV-ANOVA_* values and permutation test results (Figure 5), which can be used for a complementary metric when the *Q*_2_*Y* value is less than 0.5 [11]. Based on the results, we expect that the application of the MRS may contribute to managing the risk of dyslipidemia development but still need to improve.

## 4. Discussion

In the present study, N-acetylisoputreanine-γ-lactam and EPA were selected as a key metabolite for MRS on dyslipidemia; however, only EPA was able to be reproduced in the independent replication set. Even though N-acetylisoputreanine-γ-lactam alone did not significantly diagnose dyslipidemia in the replication set, the MRS and the combined use of the lipid profile still suggest that MRS has relevance for dyslipidemia in the Korean population. Therefore, this study demonstrated the potential of MRS as an assistive tool for dyslipidemia assessment.

N-acetylisoputreanine-γ-lactam, also known as N-(3-acetamidopropyl)pyrrolidin-2-one and acisoga, is a unique catabolite of N1-acetylspermidine [12]. This acetylated compound is one of the polyamines generated during polyamine metabolism, which originates from amino acids, arginine, and ornithine [13,14]. Spermidine/spemine-N1-acetyltransferase (SSAT), a rate-limiting enzyme essential for maintaining cellular polyamine homeostasis [14,15], plays a crucial role in polyamine metabolism by adding an acetyl group to the spermidine, thereby producing N1-acetylspermidine [14]. N1-acetylspermidine is a substrate for peroxisomal N1-acetylpolyamine oxidase (PAO), which generates H_2_O_2_ during N1-acetylspermidine degradation, resulting in oxidative stress [16,17]. Additionally, N1-acetylspermidine is also catabolized by copper-dependent amine oxidase (CuAO) and aldehyde dehydrogenase (ALDH) and converted into N-acetylisoputreanine-γ-lactam [16,17].

Whereas polyamines such as spermidine and N1-acetylspermidine have been well-studied regarding their roles in nucleic acid and protein formation, protection against oxidative stress, ion channel action, and cell survival [18,19], the exact biological functions of N-acetylisoputreanine-γ-lactam remain unclear. However, recent studies indicated its possibilities as a biomarker for heart failure, atrial fibrillation, Parkinson’s disease, and ovarian cancer, as elevated levels of N-acetylisoputreanine-γ-lactam have been observed in these conditions [20,21,22,23]. In the discovery set of the present study, N-acetylisoputreanine-γ-lactam also showed a significant increase in the dyslipidemia group, suggesting its potential as a biomarker for dyslipidemia. Unfortunately, in the replication set, the result was not validated even though the levels of this metabolite were higher in the dyslipidemia group than in the healthy group (log_2_ fold change of the dyslipidemia group relative to the healthy group in the replication set = 0.566, *q* = 0.252). There is a lack of research and evidence elucidating the associations between N-acetylisoputreanine-γ-lactam and dyslipidemia. As such, it remains uncertain whether this observation is merely by chance. One possible hypothesis is that N-acetylisoputreanine-γ-lactam is not directly or strongly related to dyslipidemia but may instead reflect potential chronic complications induced by dyslipidemia, such as the cardiovascular diseases and cancers mentioned earlier. This association needs to be further investigated through repeated studies on this polyamine metabolite in the context of dyslipidemia, given that many diseases have been shown to be linked to this metabolite.

EPA is a long-chain polyunsaturated fatty acid (PUFA) classified as ω-3 (C20:5, n-3). In humans, it is synthesized from α-linolenic acid (C18:3, n-3), an essential fatty acid that must be consumed from food [24]. However, the efficacy of EPA synthesis is very low; thus, it mostly needs to be obtained from the diet [25]. Many studies have demonstrated that EPA supplementation is beneficial to lowering blood lipids, especially in decreasing TG levels [26,27,28]. Moreover, studies have reported that the intake of ω-3 PUFAs, such as EPA, improves cardiovascular risk factors, including platelet aggregation, atherogenic lipoprotein particles, and C-reactive protein levels [29,30,31].

Regarding the EPA increment in the dyslipidemia group of the present study, although it is not possible to know for certain because of the inability to collect dietary information from the study individuals, it is thought to be due to an overall increase in fatty acid turnover rather than EPA supplementation. Indeed, not only EPA but also all other UFAs and SFAs, with the exception of capric acid (C10:0), were elevated in the dyslipidemia group. This pattern could be typical in dyslipidemia associated with obesity, as well as insulin resistance [32,33], as observed in our study population; the dyslipidemia group of the discovery set exhibited higher BMI and fasting glucose values, and even higher HbA1c levels as well [healthy group (*n* = 17): 5.427 ± 0.07, dyslipidemia group (*n* = 43): 6.542 ± 0.24, *p^a^* < 0.001, *p^b^* < 0.032]. In obesity, there is an increased flux of free fatty acids (FFAs) into the liver, where excess FFAs are used to produce TGs that are then released into the circulation [32]. Additionally, insulin resistance, accompanied by obesity, suppresses insulin from inhibiting hormone-sensitive lipase activity in adipose tissue, thereby promoting the breakdown of stored fat and releasing it as FFAs into the bloodstream [33]. The increased influx of FFAs into the liver via blood circulation perpetuates a vicious circle. In this study, only EPA was chosen as a key metabolite among the FFAs for dyslipidemia assessment. Given the focus of many studies on dietary EPA supplementation and its beneficial effects in various diseases, these results prompt consideration of the endogenous increase in EPA in disease conditions—whether it occurs naturally alongside other FFAs or through complementary mechanisms to combat such inflammatory or oxidative stress conditions.

In addition to the key metabolites, there is an overall difference in metabolite patterns between the healthy and dyslipidemia groups, particularly in ACs and amino acids. Among the ACs, short-chain ACs specifically were increased in the dyslipidemia group. The increase of these metabolites is related to a high risk of cardiovascular diseases, stroke, and type 2 diabetes [34,35,36], as dyslipidemia is also known to be associated with these conditions. The dyslipidemia group of the present study showed increased amino acid levels as well. Researchers have indicated that increased levels of branched-chain amino acids (BCAAs) are significantly related to the risk of TG level elevation and insulin resistance [37,38,39]. Moreover, BCAAs generate short-chain ACs (especially C3 and C5 ACs) as intermediate metabolites [40]. Hence, the increase in short-chain ACs and amino acids in the dyslipidemia group of this study, showing higher levels of glucose, insulin resistance (as described in the previous paragraph), and TG, is consistent with previous studies. Although the present study statistically selected only two metabolites, N-acetylisoputreanine-γ-lactam and EPA, the evidence regarding short-chain ACs and amino acids in dyslipidemia suggests that their potential applications in MRS panels are worth considering in the future.

The present study suggests the use of MRS as an assessment tool for diseases. However, it still has the limitation that MRS alone cannot yet fully screen diseases with very high diagnostic accuracy. In addition, although MRS enhanced diagnostic performance as an adjuvant tool for dyslipidemia, its practical application may not yet be timely in terms of cost-effectiveness, as metabolomics-based profiling is still required to detect the identified key metabolites. The development of point-of-care diagnostic devices or platforms capable of measuring these key metabolites in a simple and convenient manner will be crucial to enhance its practicality. Nevertheless, based on our findings, MRS holds promise as an auxiliary tool, particularly in combination with conventional markers, and this study provides a conceptual framework for its implementation in clinical settings [41]. For MRS to be applied as a professional stand-alone tool for disease diagnostics, future research should focus on expanding metabolite panels to include markers that are sensitive, specific, and reliable for dyslipidemia, as well as on establishing standardized MRS protocols. With continued advancements, MRS may ultimately overcome the limitations of current molecular diagnostic standards.

## Figures and Tables

**Table 1:** Clinical and biochemical characteristics between the healthy and dyslipidemia groups in the discovery set.

	Total (*n* = 99)				*p^a^*	*p^b^*
	Healthy (*n* = 50)		Dyslipidemia (*n* = 49)			
Age (year) *^†^*	44.8	±0.73	52.4	±0.61	<0.001	-
Male/Female *n*, (%)	33 (66.0)/17 (34.0)		36 (73.5)/13 (26.5)		0.654	
Body weight (kg) *^†^*	68.9	±1.53	76.0	±1.56	<0.001	-
BMI (kg/m^2^) *^†^*	24.5	±0.43	27.8	±0.45	<0.001	-
Systolic BP (mmHg) *^†^*	122.8	±1.57	133.1	±2.54	0.001	0.001
Diastolic BP (mmHg) *^†^*	76.2	±1.40	80.0	±1.97	0.167	0.018
Glucose (mg/dL) *^†^*	96.1	±1.38	125.4	±5.25	<0.001	0.001
TG (mg/dL) *^†^*	140.3	±11.0	175.4	±16.1	0.015	0.783
HDL-C (mg/dL)	49.0	±1.43	46.0	±2.03	0.240	0.625
LDL-C (mg/dL)	107.0	±5.84	102.2	±6.21	0.573	0.319
TC (mg/dL)	201.1	±4.66	174.6	±7.59	0.004	0.077
AST (U/L) *^†^*	23.7	±1.57	37.9	±4.72	<0.001	0.351
ALT (U/L) *^†^*	23.9	±2.00	39.3	±4.60	<0.001	0.060
TNF-α (pg/mL) *^†^*	11.0	±0.69	25.1	±2.81	<0.001	<0.001
IL-1β (pg/mL) *^†^*	4.31	±0.31	5.59	±0.43	0.008	0.103
IL-6 (pg/mL) *^†^*	2.49	±5.75	6.41	±2.83	0.002	0.064
IFN-γ (pg/mL) *^†^*	15.8	±1.28	13.4	±1.21	0.121	0.464
Ox-LDL (U/L)	49.6	±1.96	42.7	±2.07	0.017	0.515

Mean ± standard error (SE). *^†^* variables tested following logarithmic transformation. The *p^a^*-values of the continuous variables were derived from independent *t*-tests. A *p^a^*-value of the sex distribution was derived from the Chi-squared test. *p^b^*-values were *p^a^*-values that are adjusted by confounding factors, including age, body weight, and BMI. All *p* < 0.05 were considered to indicate significance. ALT: alanine aminotransferase. AST: aspartate aminotransferase. BMI: body mass index. BP: blood pressure. HDL-C: high-density lipoprotein cholesterol. IFN: interferon. IL: interleukin. LDL-C: low-density lipoprotein cholesterol. Ox: oxidized. TC: total cholesterol. TG: triglyceride. TNF: tumor necrosis factor.

## Data Availability

The data presented in this study are available on request from the corresponding author, as permission from the Biobanks is required.

## Supplementary Materials

The following supporting information can be downloaded at: https://www.mdpi.com/article/10.3390/metabo15040279/s1, Table S1: Putatively identified metabolites in both negative and positive modes within whole individuals. Table S2: Weight values (standardized β) of N-acetylisoputreanine-γ-lactam and eicosapentaenoic acid obtained through linear regression analysis in the discovery set. Table S3: Classification accuracy based on the MRS application. Figure S1: A permutation test of the positive and negative modes in the discovery set. Figure S2: VIP score plots of the top 50 metabolites observed in the positive and negative modes in the discovery set.

## Abbreviations

AC	acylcarnitine
ALT	alanine aminotransferase
AST	aspartate aminotransferase
BCAA	branched-chain amino acids
BMI	body mass index
BP	blood pressure
DPA	docosapentaenoic acid
EPA	eicosapentaenoic acid
ESI	electrospray ionization
FDR	false discovery rate
FFA	free fatty acid
HDL-C	high-density lipoprotein-cholesterol
IFN	interferon
IL	interleukin
ISTD	internal standard
KCD	Korean Standard Classification of Diseases
LDL-C	low-density lipoprotein-cholesterol
LPC	lysophosphatidylcholine
MRS	metabolite risk score
OPLS-DA	orthogonal partial least squares-discriminant analysis
ox-	oxidized
PUFA	polyunsaturated fatty acid
ROC	receiver operating characteristic
SFA	saturated fatty acid
TC	total cholesterol
TG	triglyceride
TNF	tumor necrosis factor
UFA	unsaturated fatty acid
UPLC-MS/MS	ultra-performance liquid chromatography-tandem mass spectrometry
VIP	variable importance in projection
γ-GTP	γ-glutamyltransferase

## References

[1] Jin E.S., Shim J.S., Kim S.E., Bae J.H., Kang S., Won J.C., Shin M.J., Jin H.Y., Moon J., Lee H. (2023). Dyslipidemia Fact Sheet in South Korea, 2022. Diabetes Metab. J..

[2] Berberich A.J., Hegele R.A. (2022). A modern approach to dyslipidemia. Endocr. Rev..

[3] Banach M., Surma S., Toth P.P., endorsed by the International Lipid Expert Panel (ILEP) (2023). 2023: The year in cardiovascular disease–the year of new and prospective lipid lowering therapies. Can we render dyslipidemia a rare disease by 2024?. Arch. Med. Sci..

[4] Qiu S., Cai Y., Yao H., Lin C., Xie Y., Tang S., Zhang A. (2023). Small molecule metabolites: Discovery of biomarkers and therapeutic targets. Signal Transduct. Target Ther..

[5] Du H., Rao Y., Liu R., Deng K., Guan Y., Luo D., Mao Q., Yu J., Bo T., Fan Z. (2021). Proteomics and metabolomics analyses reveal the full spectrum of inflammatory and lipid metabolic abnormalities in dyslipidemia. Biomed. Chromatogr..

[6] Ke C., Zhu X., Zhang Y., Shen Y. (2018). Metabolomic characterization of hypertension and dyslipidemia. Metabolomics.

[7] Wang N., Ru Y., Yang Z., Sun C., Li S., Min Y., Zhao X., Lu Y., Hsing A.W., Zhu S. (2021). Metabolomic profiles of plasma retinol-associated dyslipidemia in men and women. Front. Nutr..

[8] Geidenstam N., Hsu Y.H., Astley C.M., Mercader J.M., Ridderstråle M., Gonzalez M.E., Gonzalez C., Hirschhorn J.N., Salem R.M. (2019). Using metabolite profiling to construct and validate a metabolite risk score for predicting future weight gain. PLoS ONE.

[9] Gadgil M.D., Cheng J., Herrington D.M., Kandula N.R., Kanaya A.M. (2024). Adipose tissue-derived metabolite risk scores and risk for type 2 diabetes in South Asians. Int. J. Obes..

[10] He S., Granot-Hershkovitz E., Zhang Y., Bressler J., Tarraf W., Yu B., Huang T., Zeng D., Wassertheil-Smoller S., Lamar M. (2022). Blood metabolites predicting mild cognitive impairment in the study of Latinos-investigation of neurocognitive aging (HCHS/SOL). Alzheimers Dement..

[11] Ström M., Wheelock Å.M. (2024). Permutation analysis prior to variable selection greatly enhances robustness of OPLS analysis in small cohorts. bioRxiv.

[12] The Human Metabolome Database (HMDB) N-(3-Acetamidopropyl)pyrrolidin-2-One. https://hmdb.ca/metabolites/HMDB0061384.

[13] Xu H., Liu R., He B., Bi C.W., Bi K., Li Q. (2016). Polyamine metabolites profiling for characterization of lung and liver cancer using an LC-Tandem MS method with multiple statistical data mining strategies: Discovering potential cancer biomarkers in human plasma and urine. Molecules.

[14] Pegg A.E. (2008). Spermidine/spermine-*N*^1^-acetyltransferase: A key metabolic regulator. Am. J. Physiol. Endocrinol. Metab..

[15] Casero R.A., Wang Y., Stewart T.M., Devereux W., Hacker A., Wang Y., Smith R., Woster P.M. (2003). The role of polyamine catabolism in anti-tumour drug response. Biochem. Soc. Trans..

[16] Seiler N. (2004). Catabolism of polyamines. Amino Acids.

[17] Fitzgerald B.L., Mahapatra S., Farmer D.K., McNeil M.R., Casero R.A., Belisle J.T. (2017). Elucidating the structure of N1-acetylisoputreanine: A novel polyamine catabolite in human urine. ACS Omega.

[18] Pegg A.E. (2016). Functions of polyamines in mammals. J. Biol. Chem..

[19] Mandal S., Mandal A., Johansson H.E., Orjalo A.V., Park M.H. (2013). Depletion of cellular polyamines, spermidine and spermine, causes a total arrest in translation and growth in mammalian cells. Proc. Natl. Acad. Sci. USA.

[20] Puetz A., Artati A., Adamski J., Schuett K., Romeo F., Stoehr R., Marx N., Federici M., Lehrke M., Kappel B.A. (2022). Non-targeted metabolomics identify polyamine metabolite acisoga as novel biomarker for reduced left ventricular function. ESC Heart Fail..

[21] Alonso A., Yu B., Sun Y.V., Chen L.Y., Loehr L.R., O’Neal W.T., Soliman E.Z., Boerwinkle E. (2019). Serum metabolomics and incidence of atrial fibrillation (from the Atherosclerosis Risk in Communities Study). Am. J. Cardiol..

[22] LeWitt P.A., Li J., Wu K.H., Lu M. (2023). Diagnostic metabolomic profiling of Parkinson’s disease biospecimens. Neurobiol. Dis..

[23] Fahrmann J.F., Irajizad E., Kobayashi M., Vykoukal J., Dennison J.B., Murage E., Wu R., Long J.P., Do K.A., Celestino J. (2021). A MYC-driven plasma polyamine signature for early detection of ovarian cancer. Cancers.

[24] Ibarguren M., López D.J., Escribá P.V. (2014). The effect of natural and synthetic fatty acids on membrane structure, microdomain organization, cellular functions and human health. Biochim. Biophys. Acta.

[25] Jakhwal P., Kumar Biswas J., Tiwari A., Kwon E.E., Bhatnagar A. (2022). Genetic and non-genetic tailoring of microalgae for the enhanced production of eicosapentaenoic acid (EPA) and docosahexaenoic acid (DHA)—A review. Bioresour. Technol..

[26] Wang T., Zhang X., Zhou N., Shen Y., Li B., Chen B.E., Li X. (2023). Association between omega-3 fatty acid intake and dyslipidemia: A continuous dose-response meta-analysis of randomized controlled trials. J. Am. Heart Assoc..

[27] Mori T.A., Burke V., Puddey I.B., Watts G.F., O’Neal D.N., Best J.D., Beilin L.J. (2000). Purified eicosapentaenoic and docosahexaenoic acids have differential effects on serum lipids and lipoproteins, LDL particle size, glucose, and insulin in mildly hyperlipidemic men. Am. J. Clin. Nutr..

[28] Grimsgaard S., Bonaa K.H., Hansen J.B., Nordøy A. (1997). Highly purified eicosapentaenoic acid and docosahexaenoic acid in humans have similar triacylglycerol-lowering effects but divergent effects on serum fatty acids. Am. J. Clin. Nutr..

[29] Mori T.A., Woodman R.J. (2006). The independent effects of eicosapentaenoic acid and docosahexaenoic acid on cardiovascular risk factors in humans. Curr. Opin. Clin. Nutr. Metab. Care.

[30] Satoh N., Shimatsu A., Kotani K., Sakane N., Yamada K., Suganami T., Kuzuya H., Ogawa Y. (2007). Purified eicosapentaenoic acid reduces small dense LDL, remnant lipoprotein particles, and C-reactive protein in metabolic syndrome. Diabetes Care.

[31] Tani S., Nagao K., Matsumoto M., Hirayama A. (2013). Highly purified eicosapentaenoic acid may increase low-density lipoprotein particle size by improving triglyceride metabolism in patients with hypertriglyceridemia. Circ. J..

[32] Klop B., Elte J.W., Cabezas M.C. (2013). Dyslipidemia in obesity: Mechanisms and potential targets. Nutrients.

[33] Sears B., Perry M. (2015). The role of fatty acids in insulin resistance. Lipids Health Dis..

[34] Guasch-Ferré M., Zheng Y., Ruiz-Canela M., Hruby A., Martínez-González M.A., Clish C.B., Corella D., Estruch R., Ros E., Fitó M. (2016). Plasma acylcarnitines and risk of cardiovascular disease: Effect of Mediterranean diet interventions. Am. J. Clin. Nutr..

[35] Deda O., Panteris E., Meikopoulos T., Begou O., Mouskeftara T., Karagiannidis E., Papazoglou A.S., Sianos G., Theodoridis G., Gika H. (2022). Correlation of serum acylcarnitines with clinical presentation and severity of coronary artery disease. Biomolecules.

[36] Guasch-Ferré M., Ruiz-Canela M., Li J., Zheng Y., Bulló M., Wang D.D., Toledo E., Clish C., Corella D., Estruch R. (2019). Plasma acylcarnitines and risk of type 2 diabetes in a Mediterranean population at high cardiovascular risk. J. Clin. Endocrinol. Metab..

[37] Mook-Kanamori D.O., Römisch-Margl W., Kastenmüller G., Prehn C., Petersen A.K., Illig T., Gieger C., Wang-Sattler R., Meisinger C., Peters A. (2014). Increased amino acids levels and the risk of developing of hypertriglyceridemia in a 7-year follow-up. J. Endocrinol. Investig..

[38] Fukushima K., Harada S., Takeuchi A., Kurihara A., Iida M., Fukai K., Kuwabara K., Kato S., Matsumoto M., Hirata A. (2019). Association between dyslipidemia and plasma levels of branched-chain amino acids in the Japanese population without diabetes mellitus. J. Clin. Lipidol..

[39] Yang P., Hu W., Fu Z., Sun L., Zhou Y., Gong Y., Yang T., Zhou H. (2016). The positive association of branched-chain amino acids and metabolic dyslipidemia in Chinese Han population. Lipids Health Dis..

[40] Newgard C.B. (2012). Interplay between lipids and branched-chain amino acids in development of insulin resistance. Cell Metab..

[41] Yoo H.J. (2024). Application of a metabolite risk score model for dyslipidemia in Koreans (poster). 2024 ICOMES Integrating Cutting-Edge Insights in Obesity Management, Proceedings of the 2024 ICOMES (International Congress on Obesity and Metabolic Syndrome), Seoul, Republic of Korea, 5–7th September 2024.

