# Supplementary material for: A Scoring Model Using Multi-Metabolites Based on Untargeted Metabolomics for Assessing Dyslipidemia in Korean Individuals with Obesity
Source: Metabolites. 2025 Apr 17;15(4):279. doi: 10.3390/metabo15040279 (PMC12029618; doi:10.3390/metabo15040279)
Supplement: Supplementary file 1 [file metabolites-15-00279-s001.zip › metabolites-3570482-supplementary.pdf]

Supplementary information

A scoring model using multi-metabolites based on untargeted metabolomics for assessing dyslipidemia in the Korean population

Su-Geun Yang, Hye Jin Yoo

Statistical analysis

Compound Discoverer ver. 3.3 SP 2 software (Thermo Fisher, Waltham, MA, USA) was used for putative metabolite identification and acquisition of relative peak intensity data. Following this, SIMCA 17 software (Sartorius-Umetrics, Göttingen, Germany) was utilized for orthogonal partial least squares-discriminant analysis (OPLS-DA), loading plot generation, permutation tests, and VIP score analysis. Assessment of OPLS-DA model relied on R^2Y and Q^2Y values, both surpassing 0.5, denoting satisfactory goodness of fit and predictive ability, respectively. Permutation tests (100 iterations) were conducted to assess model validity and guard against overfitting; a valid model was identified if permuted R^2Y values consistently remained below the actual R^2Y values, and if the intercept of a regression line connecting permuted Q^2Y values and actual Q^2Y value was less than 0.

Statistical analysis was conducted using IBM SPSS Statistics 28.0 (IBM corp., Armonk, NY, USA). This included comparison of variables between groups, linear regression analysis, and ROC curve analysis. Independent *t*-tests were employed for group comparisons of continuous variables, while non-normally distributed variables underwent log transformation prior to analysis. Confounding factors such as age, body weight, and BMI were adjusted using analysis of covariance (ANCOVA). *Chi*-squared tests were used for group comparisons of nominal variables. Linear regression analysis was utilized to identify key metabolites, derive standardized β-coefficient values, and establish predictive models for dyslipidemia. ROC curve analysis, guided by Youden’s index, determined cut-off values of the key metabolites assessed the model predictive performance for dyslipidemia. A significance threshold of

two-tailed p <0.05 was used for all analysis. However, q-values were computed to adjust for false discovery rate (FDR) in metabolite comparison, utilizing the R software ver. 4.4.2 (R Foundation for Statistical Computing, Vienna, Austria) with package fdrtool ver. 1.2.18 [https://CRAN.R-project.org/package=fdrtool (accessed on 1st Nov. 2024)], with significance defined as q <0.05 mitigate type 1 error.

Table S1. Putatively identified metabolites in both negative and positive modes within whole individuals

Mode	*m/z*	Formula	Subclass	Name (putatively identified)	*q*	Log_2(fold change); dyslipidemia/healthy
Negative	178.050	C9 H9 N O3	Acylaminobenzoic acids	4-Acetamidobenzoic acid	0.116	1.916
Negative	180.066	C9 H11 N O3	Amino acids	L-Tyrosine	0.029	1.266
Negative	203.082	C11 H12 N2 O2	Amino acids	D-Tryptophan	0.202	-0.083
Negative	775.682	C15 H11 I4 N O4	Amino acids	Thyroxine	0.393	-0.025
Negative	284.224	C16 H31 N O3	Amino acids, peptides, and analogues	Myristoylglycine	0.013	0.189
Negative	203.002	C7 H8 O5 S	Arylsulfates	O-Methoxycatechol-O-sulphate	0.256	0.881
Negative	247.170	C16 H24 O2	Benzene and substituted derivatives	[2-(Dimethoxymethyl)-1-heptenyl]benzene	<0.001	0.720
Negative	193.123	C12 H18 O2	Benzenediols	Hexylresorcinol	0.031	-0.314
Negative	172.991	C6 H6 O4 S	Benzenesulfonic acids	4-Hydroxybenzenesulfonic acid	0.367	0.164
Negative	311.169	C17 H28 O3 S	Benzenesulfonic acids	N-Undecylbenzenesulfonic acid	0.286	-0.043
Negative	325.185	C18 H30 O3 S	Benzenesulfonic acids	4-Dodecylbenzenesulfonic acid	0.170	-0.129
Negative	369.175	C19 H30 O5 S	C19 Steroids	Androsterone sulfate	0.001	-0.169
Negative	367.159	C19 H28 O5 S	C19 Steroids	Testosterone sulfate	<0.001	-0.573
Negative	280.622	C26 H45 N O8 S2	C24 Bile acids	Taurolithocholic acid 3-sulfate	0.414	0.143
Negative	464.303	C26 H43 N O6	C24 Bile acids	Glycocholic acid	0.099	-0.269
Negative	391.286	C24 H40 O4	C24 Bile acids	Deoxycholic acid	0.326	0.336
Negative	448.308	C26 H43 N O5	C24 Bile acids	Glycoursodeoxycholic acid	0.041	-0.477
Negative	498.291	C26 H45 N O6 S	C24 Bile acids	Taurochenodesoxycholic acid	0.008	-0.397
Negative	359.296	C24 H40 O2	C24 Bile acids	5β-Cholanic acid	0.004	0.747
Negative	435.349	C27 H48 O4	C27 Bile acids	5β-cholestan-3α,7α,24,27-tetrol	0.001	-0.770
Negative	433.237	C21 H39 O7 P	Carbonyl compounds	Oleoylglycerone phosphate	0.178	-0.464
Negative	357.280	C24 H38 O2	Carbonyl compounds	1-Phenyl-1,3-octadecanedione	<0.001	2.355
Negative	199.007	C8 H8 O4 S	Cinnamic acids	4-Vinylphenol sulfate	0.043	0.448
Negative	267.124	C14 H20 O5	Heterocyclic FA	3-carboxy-4-methyl-5-pentyl-2-furanpropanoic acid	0.017	0.517
Negative	299.259	C18 H36 O3	HODA	9-Hydroxyoctadecanoic acid	0.239	0.083
Negative	311.223	C18 H32 O4	HpODE	9-Hpode	0.017	0.308
Negative	204.066	C11 H11 N O3	Indolecarboxylic acids	Indolelactic acid	0.175	0.314
Negative	277.218	C18 H30 O2	Lineolic acids and derivatives	α-Eleostearic acid	<0.001	1.202
Negative	435.252	C21 H41 O7 P	LPA	LysoPA(18:1/0:0)	0.100	-0.341
Negative	409.237	C19 H39 O7 P	LPA	LysoPA(16:0/0:0)	0.056	-0.429
Negative	463.284	C23 H45 O7 P	LPA	PA(20:1/0:0)	<0.001	-0.606
Negative	437.268	C21 H43 O7 P	LPA	LysoPA(18:0/0:0)	<0.001	-0.535
Negative	508.342	C25 H52 N O7 P	LPE	LysoPE(20:0/0:0)	<0.001	-0.607
Negative	599.322	C27 H53 O12 P	LPI	LysoPI(18:0/0:0)	0.127	-0.128
Negative	327.255	C19 H36 O4	MAG	MG(16:1/0:0/0:0)	0.270	-0.105
Negative	201.022	C8 H10 O4 S	Other phenols	4-Ethylphenylsulfate	0.137	0.768
Negative	239.067	C10 H12 N2 O5	Other phenols	2-(1-Methylpropyl)-4,6-dinitrophenol	0.354	-0.030
Negative	738.510	C41 H74 N O8 P	PE	PE(16:0/20:4)	<0.001	-0.389
Negative	187.006	C7 H8 O4 S	Phenylsulfates	p-Cresol sulfate	0.346	0.038

Negative	243.062	C9 H12 N2 O6	Pyrimidine ribonucleosides	Uridine	0.463	-0.013
Negative	171.138	C10 H20 O2	Saturated FA	Capric acid	0.002	-0.419
Negative	199.170	C12 H24 O2	Saturated FA	Dodecanoic acid	0.204	0.181
Negative	227.201	C14 H28 O2	Saturated FA	Myristic acid	<0.001	0.951
Negative	241.217	C15 H30 O2	Saturated FA	Pentadecanoic acid	0.004	0.487
Negative	269.249	C17 H34 O2	Saturated FA	Heptadecanoic acid	<0.001	0.742
Negative	283.265	C18 H36 O2	Saturated FA	Stearic acid	<0.001	0.664
Negative	255.233	C16 H32 O2	Saturated FA	Palmitic acid	0.011	0.375
Negative	212.002	C8 H7 N O4 S	Simple indole alkaloids	Indoxyl sulfate	0.108	0.346
Negative	465.250	C25 H38 O8	Steroidal glycosides	5α-Dihydrotestosterone glucuronide	0.404	1.253
Negative	191.019	C6 H8 O7	TCA acids	Isocitric acid	0.459	-0.055
Negative	183.138	C11 H20 O2	Unsaturated FA	Undecylenic acid	0.447	-0.037
Negative	223.170	C14 H24 O2	Unsaturated FA	Goshuyic acid	0.104	0.109
Negative	225.186	C14 H26 O2	Unsaturated FA	Myristoleic acid	<0.001	0.691
Negative	275.202	C18 H28 O2	Unsaturated FA	Stearidonic acid	<0.001	1.895
Negative	301.218	C20 H30 O2	Unsaturated FA	Eicosapentaenoic acid	<0.001	1.931
Negative	253.217	C16 H30 O2	Unsaturated FA	Palmitoleic acid	<0.001	1.134
Negative	327.233	C22 H32 O2	Unsaturated FA	Docosahexaenoic acid	<0.001	1.454
Negative	279.233	C18 H32 O2	Unsaturated FA	Linoelaidic acid	<0.001	0.833
Negative	303.233	C20 H32 O2	Unsaturated FA	Arachidonic acid	<0.001	1.059
Negative	267.233	C17 H32 O2	Unsaturated FA	Trans-10-Heptadecenoic acid	<0.001	1.054
Negative	329.249	C22 H34 O2	Unsaturated FA	Docosapentaenoic acid	<0.001	1.704
Negative	305.249	C20 H34 O2	Unsaturated FA	Dihomo-γ-linolenic acid	<0.001	1.419
Negative	281.249	C18 H34 O2	Unsaturated FA	Oleic acid	<0.001	0.849
Negative	307.265	C20 H36 O2	Unsaturated FA	Eicosadienoic acid	<0.001	1.020
Negative	309.280	C20 H38 O2	Unsaturated FA	Eicosenoic acid	<0.001	1.213
Negative	365.343	C24 H46 O2	Unsaturated FA	Nervonic acid	0.167	0.634
Negative	295.265	C19 H36 O2	Wax diesters	Methyl oleate	<0.001	0.746
Negative	255.233	C16 H32 O2	Wax monoesters	Ethyl tetradecanoate	<0.001	0.845
Negative	167.020	C5 H4 N4 O3	Xanthines	Uric acid	0.425	0.040
Positive	204.123	C9 H17 N O4	Acyl carnitines	L-Acetylcarnitine	0.305	0.305
Positive	218.138	C10 H19 N O4	Acyl carnitines	Propionylcarnitine	0.332	0.133
Positive	262.165	C12 H23 N O5	Acyl carnitines	3-Hydroxyisovalerylcarnitine	0.427	0.239
Positive	232.154	C11 H21 N O4	Acyl carnitines	Butyrylcarnitine	0.175	0.331
Positive	246.170	C12 H23 N O4	Acyl carnitines	2-Methylbutyroylcarnitine	0.043	-0.259
Positive	260.185	C13 H25 N O4	Acyl carnitines	Hexanoylcarnitine	0.374	0.171
Positive	304.212	C15 H29 N O5	Acyl carnitines	3-hydroxyoctanoylcarnitine	0.403	0.186
Positive	286.201	C15 H27 N O4	Acyl carnitines	2-Octenoylcarnitine	0.003	-0.362
Positive	288.217	C15 H29 N O4	Acyl carnitines	Octanoylcarnitine	<0.001	-0.461
Positive	314.232	C17 H31 N O4	Acyl carnitines	9-Decenoylcarnitine	0.003	-0.329

Positive	316.248	C17 H33 N O4	Acyl carnitines	Decanoylcarnitine	0.002	-0.513
Positive	342.263	C19 H35 N O4	Acyl carnitines	Trans-2-Dodecenoylcarnitine	0.457	0.131
Positive	386.289	C21 H39 N O5	Acyl carnitines	3-Hydroxy-cis-5-tetradecenoylcarnitine	0.399	0.009
Positive	368.279	C21 H37 N O4	Acyl carnitines	3, 5-Tetradecadiencarnitine	0.403	0.048
Positive	344.279	C19 H37 N O4	Acyl carnitines	Dodecanoylcarnitine	0.026	-0.333
Positive	414.321	C23 H43 N O5	Acyl carnitines	3-Hydroxyhexadecenoylcarnitine	0.144	0.167
Positive	370.295	C21 H39 N O4	Acyl carnitines	cis-5-Tetradecenoylcarnitine	0.455	0.018
Positive	396.310	C23 H41 N O4	Acyl carnitines	9,12-Hexadecadienoylcarnitine	0.432	0.038
Positive	372.310	C21 H41 N O4	Acyl carnitines	Tetradecanoylcarnitine	0.005	-0.303
Positive	398.326	C23 H43 N O4	Acyl carnitines	Trans-2-hexadecenoylcarnitine	0.085	-0.210
Positive	424.341	C25 H45 N O4	Acyl carnitines	Linoleyl carnitine	<0.001	-0.835
Positive	400.341	C23 H45 N O4	Acyl carnitines	Palmitoylcarnitine	<0.001	-0.543
Positive	426.357	C25 H47 N O4	Acyl carnitines	Oleoylcarnitine	<0.001	-0.449
Positive	428.373	C25 H49 N O4	Acyl carnitines	Stearoylcarnitine	<0.001	-0.582
Positive	456.404	C27 H53 N O4	Acyl carnitines	Arachidyl carnitine	<0.001	-0.709
Positive	540.498	C33 H65 N O4	Acyl carnitines	Hexacosanoyl carnitine	<0.001	-0.718
Positive	130.159	C8 H19 N	Amines	Diisopropylethylamine	0.403	-0.067
Positive	147.113	C6 H14 N2 O2	Amino acids	Lysine	0.179	0.071
Positive	156.077	C6 H9 N3 O2	Amino acids	Histidine	0.295	-0.135
Positive	175.119	C6 H14 N4 O2	Amino acids	DL-Arginine	0.422	0.026
Positive	118.086	C5 H11 N O2	Amino acids	Betaine	0.400	0.004
Positive	132.077	C4 H9 N3 O2	Amino acids	Creatine	0.467	0.079
Positive	116.071	C5 H9 N O2	Amino acids	D-Proline	0.294	-0.057
Positive	147.076	C5 H10 N2 O3	Amino acids	Glutamine	0.387	-0.037
Positive	230.096	C9 H15 N3 O2 S	Amino acids	Ergothioneine	0.223	-0.248
Positive	130.086	C6 H11 N O2	Amino acids	D-Pipecolic acid	0.258	-0.030
Positive	132.102	C6 H13 N O2	Amino acids	Isoleucine	0.100	0.174
Positive	132.102	C6 H13 N O2	Amino acids	Leucine	0.250	0.099
Positive	220.118	C9 H17 N O5	Amino acids	Pantothenic acid	0.142	0.185
Positive	265.118	C13 H16 N2 O4	Amino acids	Phenylacetylglutamine	0.069	1.735
Positive	130.050	C5 H7 N O3	Amino acids	Pyroglutamic acid	0.001	1.407
Positive	203.150	C8 H18 N4 O2	Amino acids, peptides, and analogues	Ethyl argininate	0.096	0.327
Positive	232.154	C11 H21 N O4	Amino acids, peptides, and analogues	Tert-Butoxycarbonyl-L-leucine	0.283	0.199
Positive	197.128	C10 H16 N2 O2	Amino acids, peptides, and analogues	Cyclo(L-prolyl-L-valyl)	0.102	0.151
Positive	448.341	C27 H45 N O4	Amino acids, peptides, and analogues	N-Stearoyl tyrosine	<0.001	-1.022
Positive	340.284	C20 H37 N O3	Amino acids, peptides, and analogues	N-Oleoylglycine	<0.001	0.680
Positive	136.039	C7 H5 N O2	Benzoxazolones	2-Benzoxazolol	0.245	0.372
Positive	585.270	C33 H36 N4 O6	Bilirubins	Bilirubin	0.173	-0.175
Positive	363.217	C21 H30 O5	C21 Steroids	Cortisol	0.159	0.082
Positive	387.252	C24 H34 O4	C24 Bile acids	12α-hydroxy-3-oxocholadienic acid	0.008	0.617

Positive	565.404	C40 H52 O2	C40 isoprenoids	ε,ε-Carotene-3,3'-dione	0.261	0.032
Positive	89.060	C4 H8 O2	Carboxylic acids	Ethyl acetate	<0.001	-2.239
Positive	162.112	C7 H15 N O3	Carnitines	DL-Carnitine	0.436	0.003
Positive	344.315	C20 H41 N O3	Ceramides	N-acetylsphinganine	0.438	-0.045
Positive	104.107	C5 H13 N O	Cholines	Choline	0.404	0.057
Positive	133.032	C5 H8 O2 S	Dihydrothiophenes	3-Methylsulfolene	0.446	-0.124
Positive	229.154	C11 H20 N2 O3	Dipeptides	Leucylproline	0.173	1.014
Positive	247.129	C10 H18 N2 O5	Dipeptides	Aspartyl-Leucine	0.167	0.237
Positive	263.139	C14 H18 N2 O3	Dipeptides	Phenylalanylproline	0.278	-0.064
Positive	203.139	C9 H18 N2 O3	Dipeptides	Alanylisoleucine	<0.001	1.089
Positive	352.165	C20 H21 N3 O3	Dipeptides	Tryptophyl-Phenylalanine	<0.001	-0.328
Positive	288.155	C12 H21 N3 O5	Dipeptides	Methyl N-acetyl-L-alanyl-L-alanyl-L-alaninate	<0.001	-0.307
Positive	255.210	C19 H26	Diterpenoids	18-Nor-4(19),8,11,13-abietatetraene	0.399	0.109
Positive	207.159	C10 H22 O4	Ethers	Triethylene glycol monobutyl ether	<0.001	-2.153
Positive	422.326	C25 H43 N O4	Fatty acid esters	α-Linolenylcarnitine	<0.001	-0.564
Positive	442.352	C25 H47 N O5	Fatty acid esters	3-Hydroxyoctadecenoylcarnitine	0.312	0.001
Positive	444.367	C25 H49 N O5	Fatty acid esters	12-Hydroxy-12-octadecanoylcarnitine	<0.001	-0.407
Positive	328.248	C18 H33 N O4	Fatty acids and conjugates	(9E)-9-Nitrooctadecenoic Acid	0.302	-0.062
Positive	101.096	C6 H12 O	Fatty aldehydes	Hexanal	<0.001	-2.319
Positive	280.263	C18 H33 N O	Fatty amides	9,12-Octadecadienamide	0.001	1.169
Positive	84.081	C5 H9 N	Hydropyridines	1-Piperideine	0.460	-0.009
Positive	114.066	C4 H7 N3 O	Imidazolines	Creatinine	0.224	0.475
Positive	192.065	C10 H9 N O3	Indoleacetic acids	5-Hydroxyindoleacetic acid	0.243	0.199
Positive	188.070	C11 H9 N O2	Indoles	Trans-3-Indoleacrylic acid	0.387	-0.200
Positive	542.324	C28 H48 N O7 P	LPC	LysoPC(20:5/0:0)	0.170	0.277
Positive	468.308	C22 H46 N O7 P	LPC	LysoPC(14:0/0:0)	<0.001	-0.378
Positive	518.324	C26 H48 N O7 P	LPC	LysoPC(18:3/0:0)	0.219	-0.043
Positive	494.323	C24 H48 N O7 P	LPC	LysoPC(16:1/0:0)	0.140	-0.056
Positive	520.339	C26 H50 N O7 P	LPC	LysoPC(0:0/18:2)	0.001	-0.273
Positive	568.339	C30 H50 N O7 P	LPC	LysoPC(22:6/0:0)	0.289	0.378
Positive	544.339	C28 H50 N O7 P	LPC	LysoPC(20:4/0:0)	0.140	-0.023
Positive	520.339	C26 H50 N O7 P	LPC	LysoPC(18:2/0:0)	<0.001	-0.289
Positive	570.354	C30 H52 N O7 P	LPC	LysoPC(22:5/0:0)	0.318	0.402
Positive	496.339	C24 H50 N O7 P	LPC	LysoPC(0:0/16:0)	<0.001	-0.258
Positive	522.354	C26 H52 N O7 P	LPC	LysoPC(18:1/0:0)	0.069	-0.357
Positive	572.370	C30 H54 N O7 P	LPC	LysoPC(22:4/0:0)	0.001	-0.204
Positive	482.360	C24 H52 N O6 P	LPC	LysoPC(O-16:0/0:0)	<0.001	-0.752
Positive	510.355	C25 H52 N O7 P	LPC	LysoPC(17:0/0:0)	<0.001	-0.501
Positive	524.370	C26 H54 N O7 P	LPC	Platelet-activating factor	<0.001	-0.345
Positive	508.376	C26 H54 N O6 P	LPC	LysoPC(P-18:0/0:0)	<0.001	-0.896

Positive	510.392	C26 H56 N O6 P	LPC	LysoPC(O-18:0/0:0)	<0.001	-0.661
Positive	500.276	C25 H42 N O7 P	LPE	LypoPE(20:5/0:0)	0.002	0.741
Positive	478.292	C23 H44 N O7 P	LPE	LysoPE(18:2/0:0)	0.044	-0.209
Positive	482.323	C23 H48 N O7 P	LPE	LysoPE(0:0/18:0)	<0.001	-0.578
Positive	502.292	C25 H44 N O7 P	LPE	LysoPE(0:0/20:4)	0.441	0.174
Positive	508.339	C25 H50 N O7 P	LPE	LysoPE(0:0/20:1)	0.123	-0.092
Positive	454.292	C21 H44 N O7 P	LPE	LysoPE(16:0/0:0)	0.023	-0.170
Positive	438.297	C21 H44 N O6 P	LPE	LypoPE(P-16:0/0:0)	<0.001	-0.646
Positive	526.292	C27 H44 N O7 P	LPE	LysoPE(22:6/0:0)	<0.001	-0.597
Positive	300.289	C18 H37 N O2	NAE	Palmitoylethanolamide	0.457	-0.055
Positive	326.305	C20 H39 N O2	NAE	Oleoylethanolamide	0.247	0.097
Positive	185.128	C9 H16 N2 O2	N-alkylpyrrolidines	N-Acetylisoputreanine-γ-lactam	0.002	0.885
Positive	550.386	C28 H56 N O7 P	O-PC	PC(O-18:1/2:0)	<0.001	-0.428
Positive	270.279	C17 H35 N O	Other carboxylic acids	Capsiamide	0.146	-0.124
Positive	126.091	C7 H11 N O	Oxazoles	5-Butyloxazole	<0.001	0.768
Positive	792.553	C45 H78 N O8 P	PC	PC(15:0/22:6)	<0.001	-0.715
Positive	758.569	C42 H80 N O8 P	PE-NMe	PE-NMe(18:1/18:1)	<0.001	-0.237
Positive	123.044	C7 H6 O2	Phenolic acids	4-Hydroxybenzaldehyde	0.449	-0.072
Positive	107.049	C7 H6 O	Phenolic acids	Benzaldehyde	0.216	0.064
Positive	352.224	C16 H34 N O5 P	Phosphate esters	Sphingosine 1-phosphate (d16:1-P)	<0.001	-0.877
Positive	703.574	C39 H79 N2 O6 P	Phosphosphingolipids	SM(d18:1/16:0)	<0.001	-0.252
Positive	316.284	C18 H37 N O3	Phytosphingosines	Dehydrophytosphingosine	0.437	-0.017
Positive	86.097	C5 H11 N	Piperidine alkaloids	Piperidine	0.146	0.172
Positive	286.143	C17 H19 N O3	Piperidine alkaloids	Piperine	<0.001	-0.864
Positive	283.175	C12 H26 O7	Polyethylene glycols	Hexaethylene glycol	<0.001	-1.574
Positive	327.201	C14 H30 O8	Polyethylene glycols	Heptaethylene glycol	<0.001	-0.824
Positive	195.088	C8 H10 N4 O2	Purines and purine derivatives	1,3,9-Trimethylxanthine	0.013	-0.827
Positive	100.076	C5 H9 N O	Pyrrolidones	2-Pyrrolidinone	0.425	0.148
Positive	269.226	C20 H28	Sesquiterpenoids	Anhydroretinol	0.086	-0.184
Positive	145.122	C8 H16 O2	Short fatty esters	Butyl butyrate	<0.001	-2.240
Positive	146.060	C9 H7 N O	Simple indole alkaloids	1H-Indole-4-carbaldehyde	0.305	-0.160
Positive	302.305	C18 H39 N O2	Sphinganines	Sphinganine	<0.001	-0.947
Positive	380.255	C18 H38 N O5 P	Sphingoid base 1-P	Sphingosine-1-phosphate	<0.001	-0.961
Positive	382.271	C18 H40 N O5 P	Sphingoid base 1-P	Sphinganine 1-phosphate	<0.001	-1.310
Positive	300.289	C18 H37 N O2	Sphingosines	Sphingosine	<0.001	-0.605
Positive	427.357	C29 H46 O2	Triterpenoids	4β-Methylzymosterol-4-carbaldehyde	0.395	0.128
Positive	181.072	C7 H8 N4 O2	Xanthines	Theobromine	0.005	-0.982
Positive	181.072	C7 H8 N4 O2	Xanthines	Paraxanthine	0.025	-0.807

Table S2. Weight values (standardized *β*) of N-acetylisoputreanine-γ-lactam and eicosapentaenoic acid obtained through linear regression analysis in the discovery set.

Independent variables	*β*	Standardized *β*	*p*	CI	Significance *F*
N-Acetylisoputreanine-γ-lactam	0.121	0.312	<0.001	0.059-0.182	<0.001
Eicosapentaenoic acid	0.276	0.520	<0.001	0.192-0.360	

CI: confidence interval. The weight values locked down and applied to the replication set.

Table S3. Classification accuracy based on the MRS application

		Replication set		Total	*p*
		Healthy	Dyslipidemia		
**Re-grouped replication set**	**Healthy** *n*, (%)	31 (77.5)	9 (22.5)	40	**<0.001**
	**Dyslipidemia** *n*, (%)	19 (31.7)	41 (68.3)	60	
	**Total**	50	50	100	

p-Value was derived from *Chi-squared test.*

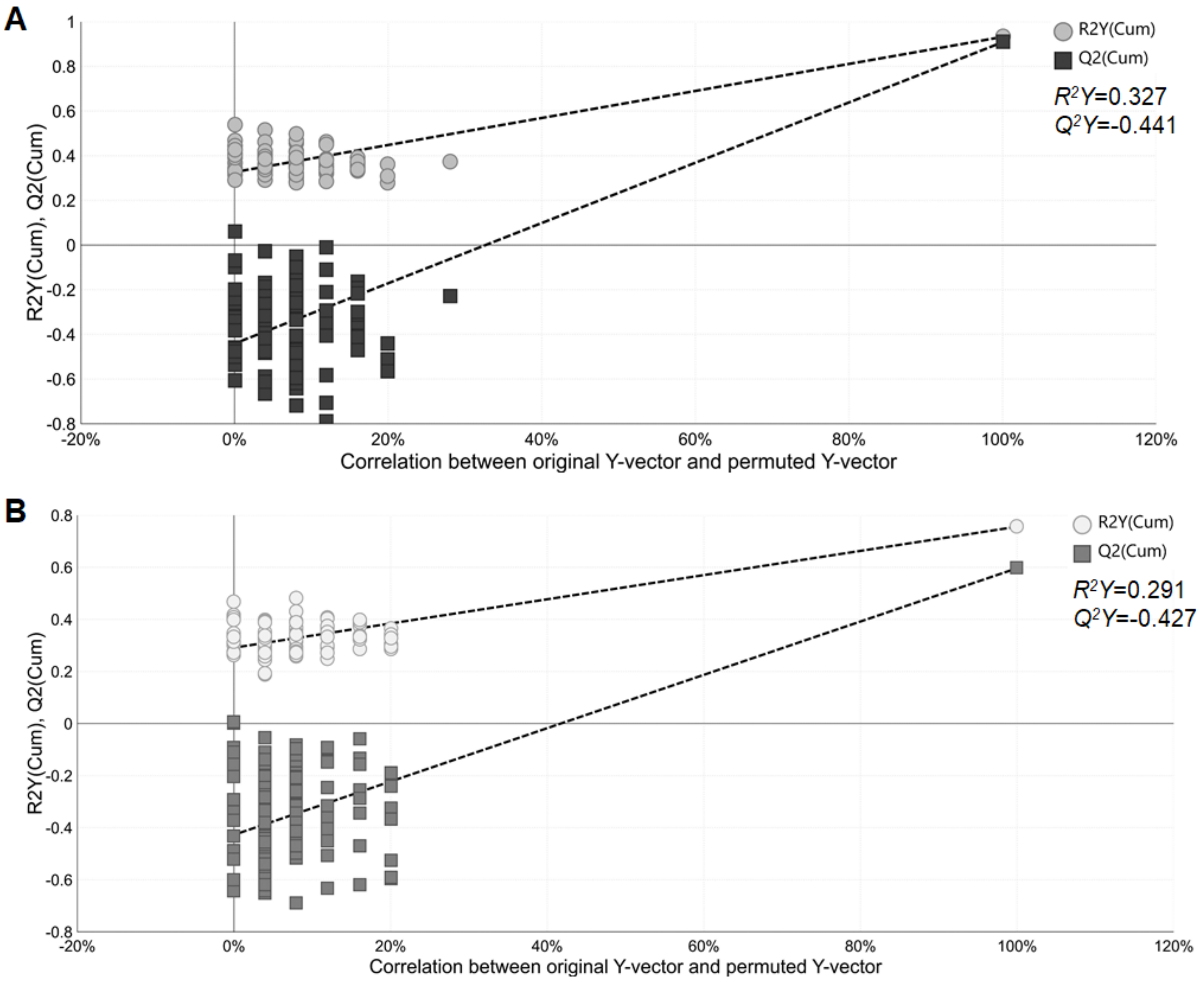

Figure S1. A permutation test of the positive and negative modes in the discovery set.

100 permutation tests were performed in both modes of the discovery set. (A) Positive mode. (B) Negative mode.

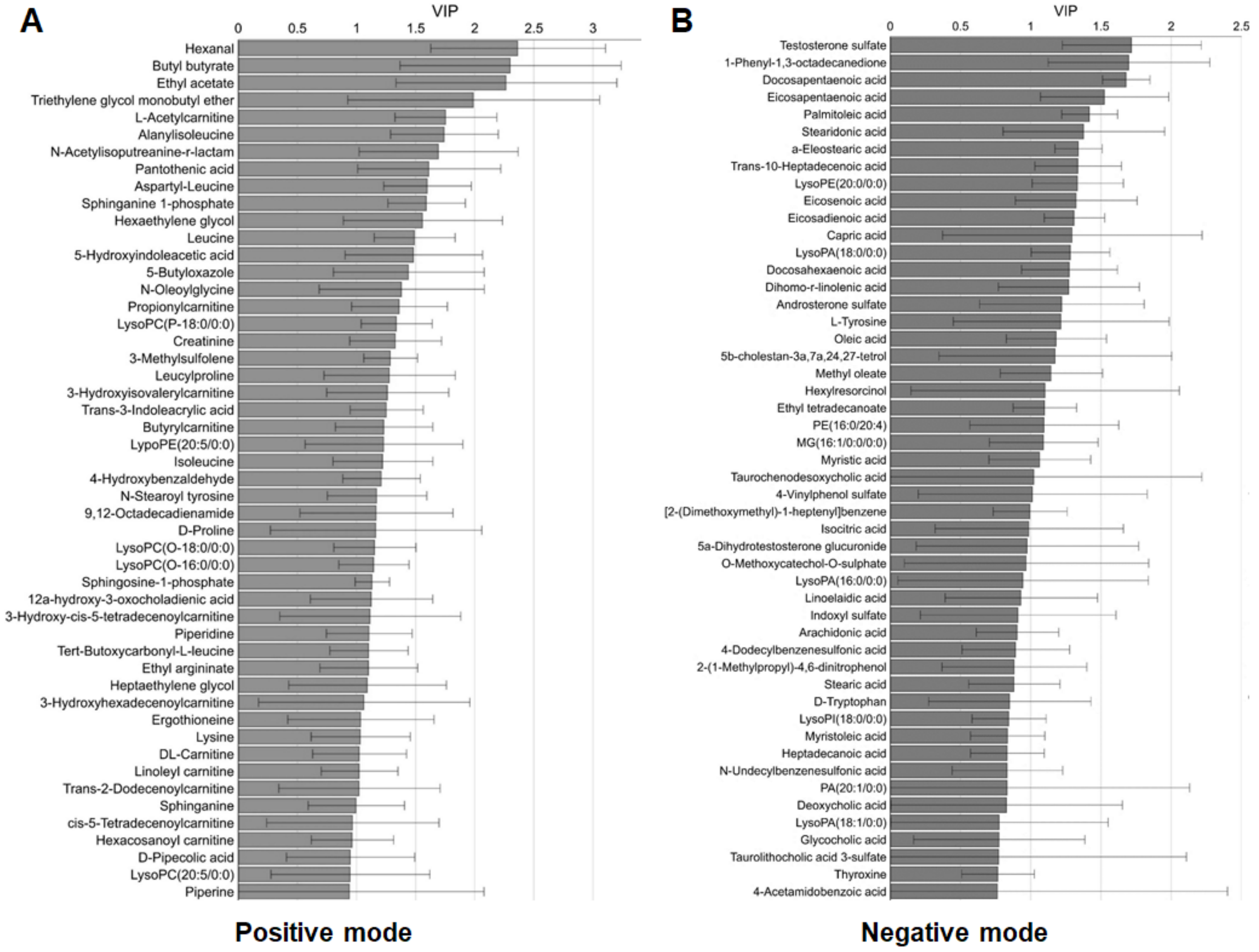

Figure S2. VIP score plots of the top 50 metabolites observed in the positive and negative modes in the discovery set.

(A) The top 50 metabolites in the positive mode. A total of 44 metabolites have VIP values over 1.0, of which 11 have VIP values exceeding 1.5. **(B)** The top 50 metabolites in the negative mode. A total of 38 metabolites have VIP values over 1.0, of which 4 have VIP values exceeding 1.5.
